# PatentMatrix: an automated tool to survey patents related to large sets of genes or proteins

**DOI:** 10.1186/1751-0473-2-4

**Published:** 2007-09-06

**Authors:** Armin Lahm, Emanuele de Rinaldis

**Affiliations:** 1 Biotechnology Department, IRBM P. Angeletti, Merck MRL-Rome, Via Pontina Km, 30600 Pomezia, Italy

## Abstract

**Background:**

The number of patents associated with genes and proteins and the amount of information contained in each patent often present a real obstacle to the rapid evaluation of the novelty of findings associated to genes from an intellectual property (IP) perspective. This assessment, normally carried out by expert patent professionals, can therefore become cumbersome and time consuming. Here we present PatentMatrix, a novel software tool for the automated analysis of patent sequence text entries.

**Methods and Results:**

PatentMatrix is written in the Awk language and requires installation of the Derwent GENESEQ™ patent sequence database under the sequence retrieval system SRS.

The software works by taking as input two files: i) a list of genes or proteins with the associated GENESEQ™ patent sequence accession numbers ii) a list of keywords describing the research context of interest (e.g. 'lung', 'cancer', 'therapeutics', 'diagnostics'). The GENESEQ™ database is interrogated through the SRS system and each patent entry of interest is screened for the occurrence of user-defined keywords. Moreover, the software extracts the basic information useful for a preliminary assessment of the IP coverage of each patent from the GENESEQ™ database. As output, two tab-delimited files are generated which provide the user with a detailed and an aggregated view of the results.

An example is given where the IP position of five genes is evaluated in the context of 'development of antibodies for cancer treatment'

**Conclusion:**

PatentMatrix allows a rapid survey of patents associated with genes or proteins in a particular area of interest as defined by keywords. It can be efficiently used to evaluate the IP-related novelty of scientific findings and to rank genes or proteins according to their IP position.

## Background

The advent of high throughput technologies for the parallel analysis of large groups of genes and proteins has challenged bioinformatics with diverse analytical needs to exploit and interpret the large volume of data produced [1] Many commercial and freeware software packages are available to assist the various phases of generating and analyzing genomics and proteomics data; from data acquisition through processing, integration and final data interpretation [2]. The results usually converge in one list of genes or proteins observed to share a common feature like differential regulation, expression pattern and cellular localization.

To assess the scientific novelty of the results obtained – e.g. a list of genes found to be differentially expressed in liver cancer – a number of publicly available text mining tools, such as PubMatrix [3] or MedMiner [4] can be used to explore the scientific literature in a systematic way.

PubMatrix is a web-based tool for text-based mining of the NCBI literature search service PubMed [5], finding associations between one or more gene names and a list of relevant research area keywords such as 'cancer', 'liver', 'over-expressed'. Co-occurrence of a gene's name with specific keywords indicates a possible connection that can be further explored.

To date no equivalent software tools are publicly available to assist researchers in assessing the novelty of findings from the viewpoint of intellectual property (IP) and thus a detailed study of many patents related to each gene or protein in the list is generally necessary. Given the large number of patents usually associated with genes and proteins and the amount of information contained in each patent entry, this is a cumbersome and time consuming activity that needs to be carried out by expert patent professionals. PatentMatrix aims at facilitating a preliminary patent overview by applying a PubMatrix-like approach to the proprietary GENESEQ™ patent sequence database (provided by Derwent Thomson Scientific [6]. The patent entries are accessed through the Sequence Retrieval System (SRS, provided by Biowisdom [7]) and screened for the occurrence of user-defined keywords. In this way it is possible to identify connections between the genes/proteins described in patents and a specific research context as defined by the user-defined keywords. The software also extracts from each GENESEQ™ entry the basic information for a preliminary assessment of the gene/protein IP status. The user is presented with a 'standard' and an 'aggregated' output providing respectively a detailed and a more concise overview – grouped by gene – of the patent analysis results.

## Methods

The software is currently designed to analyze patent entries from the GENESEQ™ database installed under the sequence retrieval system SRS. SRS is one of the most highly utilized bioinformatic platforms for data integration, analysis and display of genomics and related data. It is widely adopted in both academic and industrial scientific environments (an updated list of currently available public SRS servers can be found in [8]). The GENESEQ™ database is mostly used in industrial environments and contains information on nucleic acid and protein sequences extracted from Derwent World Patents Index basic patent documents published by 41 patent offices worldwide. Coverage includes nucleotide sequences of 10 or more bases, all amino acid sequences of 4 or more residues and nucleic probes and primers of any length. GENESEQ™ contains nucleic and amino acid sequence information associated with the first publication of each patent. In addition to sequence information, each GENESEQ™ entry contains structured textual information describing the invention and many related items such as the patent assignee, the patent status (e.g. 'pending', 'granted' etc.), the country code, the keywords associated with the invention and many other information.

The general idea behind PatentMatrix is that of screening the GENESEQ™ patent entries to find all the known (and IP protected) relationships between each gene/protein and a specific research area as described in a list of user-defined keywords. Moreover, the software extracts the basic information useful for a preliminary assessment of the patent's coverage, from an IP viewpoint from the GENESEQ™ database. The basic principles which guided the design of PatentMatrix can be summarized as follows:

1. patents are less or more relevant with respect to a specific research area or application (e.g. the exploitation of a gene as therapeutic target for liver cancer) depending on the presence of crucial keywords within the patent entry (e.g. "liver", "cancer", "therapy", "antibody").

2. "granted" patents are "stronger" than patents whose formal acceptance is still pending (e.g. patents reported in the database as "application", "unexamined", "granted unexamined" etc.).

3. patents are "stronger" if the gene or the protein sequence appears in the claims, because the invention also covers identification of the sequence and not only a specific application.

4. patents are "stronger" the larger the geographic area the application covers.

**Figure 1:**
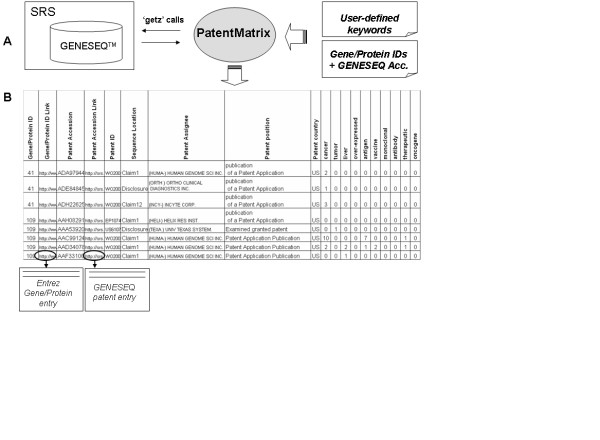
**A **Software architecture. PatentMatrix takes as input two files: i) a list of genes or proteins with the associated patent sequence accession numbers ii) a list of keywords describing the research context of interest. Each IP entry is retrieved from the GENESEQ™ database through 'getz' calls to the SRS system. **B **'standard' output file. The 'standard' output is a tab delimited table displaying for each IP entry the results of the analysis. Hyperlinks to the Entrez Gene to the GENESEQ™ databases are available. The individual output fields are described in the "Methods" section.

PatentMatrix requires two files as input: i) a list of gene or protein identifiers and associated patent sequence accession numbers ii) a list of user-defined keywords (Figure 1A). In order to identify the patent entries associated with each gene or protein to be listed in the first input file, methods based on sequence similarity such as BLAST, FASTA or others can be used [9]. The usage of our software requires that one first retrieves the patent sequence accession numbers associated with the list of gene/protein identifiers, using one of the methods mentioned above.

Examples of formats for the two input files are listed below:

Input file 1 (genes/proteins of interest and related patent identifiers):

Gene/Protein ID Patent Accession Number

41 AAC59252

41 ABQ87864

109 ACD76577

109 ABB78956

... ...

Input file 2 (user defined keywords):

liver

cancer

therapy

antibody

...

All patent entries from the input list of input file 1 are retrieved from the GENESEQ™ database and screened for each of the keywords provided in input file 2. During parsing the position of text containing the gene/protein sequence within the patent entry is also checked (e.g. in "claims", disclosure", "examples"). Moreover, information describing the applicant's name, applicant country and the patent status (e.g. "pending", "granted" etc.) is extracted. This information can be very useful for a preliminary assessment of the gene's IP status, according to points 2, 3 and 4 illustrated above.

A matrix is then generated ('standard' output file) with records of individual patent sequence accession numbers, annotated with the following fields (Fig. 1B):

- Gene/Protein ID: identifier of the gene/protein

- Gene/Protein ID Link: hyperlink to the Entrez gene/protein entry

- Patent Accession: accession number of the GENESEQ™ entry

- Patent Accession Link: hyperlink to the SRS GENESEQ™ entry

- Patent ID: official patent identifier

- Sequence Location: textual position of the gene/protein sequence within the GENESEQ™ entry (e.g. "claim", disclosure", "example")

- Patent Assignee: the entity (person, company, institution) by whom the patent was submitted

- Patent status: e.g. "granted", "application", "unexamined" etc.

- Patent country: indicates the geographic coverage of the patent (e.g. Europe, US, etc.)

In addition to the fields listed above, a column is added for each of the user-defined keywords provided as input. These columns describe the occurrences of each input keyword in the patent entry description.

**Figure 2:**
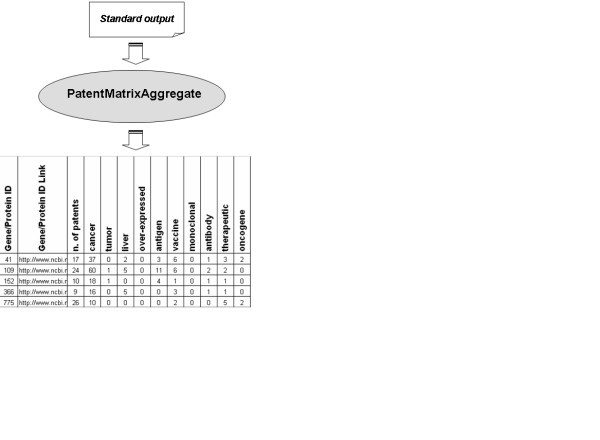
'aggregated' output file. PatentMatrixAggregate is used to aggregate the 'standard' output allowing a global "gene centric" view of the IP status. For each gene/protein the total number of keywords found in the associated patent entries is displayed.

An 'aggregated' second output file can be generated by using an auxiliary script called PatentMatrixAggregate. The 'aggregated' output combines the results from all the associated patents and keywords for each gene/protein in the input list, thus providing the user with a more concise overview of the results (Fig. 2).

## Results and Discussion

The diagram in Fig. 1A illustrates the data flow: the software takes as input two files from the command line: i) a list of gene or protein identifiers and their related patent sequence accession numbers ii) a list of user-defined keywords. The software then performs queries to the GENESEQ™ database through SRS [10] using the SRS command line query language 'getz'. For each record of the first input file, the corresponding patent entry in SRS is accessed and examined as illustrated above. It is important to notice that the GENESEQ™ database is designed in a sequence-centered manner and each patent entry refers to an individual nucleic or amino acid sequence. This implies that if one patent reports different sequences, this translates into different GENESEQ™ patent entries. Therefore all the information extracted from a GENESEQ™ patent entry can be unambiguously referred to the associated gene or protein sequence.

A tab delimited file containing the results of the analysis for each gene/protein patent entry is generated as standard output. The aggregated output (described in previous section) can be generated by using the auxiliary script 'PatentMatrixAggregate'.

In addition to keyword counts, measures such as odd ratios or p-values could be utilized to represent the deviation of keyword occurrence from average values calculated over the entire database, or in a random sample. For example, if the word "cancer" is found 20 times associated to gene X and the average count over the entire patent database is 22, the – count does not deviate much from the average. Nevertheless, the observed count of 20 indicates that for gene X there is probably already IP related to cancer. We have therefore chosen to directly report the keyword count, leaving the task of evaluating the results to the user according to individual criteria. This measure, although simple, can be very effective in singling out those genes or proteins from a long list for which there is only limited IP information for a role in a given area of research.

As the semantic linking the keywords with genes/proteins in each patent entry is not taken into account, a number of false positives is to be expected. It is therefore important to emphasize that the information provided by PatentMatrix is not intended as conclusive or as a substitute for an expert and detailed evaluation of the single patent entries. However, we believe this type of general IP statistics to be very useful in prioritizing a long list of genes/proteins for "patent novelty" in a given area of research. Our software is designed to analyze the patents provided as input in a sequential manner, and can be scaled up linearly, with virtually no limits to the length or number of input patents. However, we envisage PatentMatrix as being normally used with a number of patents related to between 50 and 1,000 genes/proteins, a typical result list of a proteomics (e.g. LC-MS) or genomics (e.g. DNA microarray) study.

PatentMatrix is written in the Awk scripting language [11] and requires a UNIX machine where an SRS installation, including the GENESEQ™ database, is present. This tool is available on request from the authors, and the package also includes examples and full documentation. As Awk is a standard Unix interpreted script language, PatentMatrix is fully compatible with any Unix system and does not require any specific installation procedure. A future development of PatentMatrix could be access through a graphical user interface (GUI) to improve its user-friendliness. However, an important advantage of keeping the software in the command-line version lies in the ease of integration with other software applications, especially from the perspective of using this tool as part of a more complex workflow for the analysis of collections of genes/proteins.

### Empirical demonstration

To briefly illustrate the capabilities of PatentMatrix, we describe the analysis results derived from a short list of genes (gene IDs: '41', '109', '152', '366','775', respectively referring to gene names ACCN2, ADCY3, ADRA2C, AQP9, CACNA1C) for which a total number of 99 patent entries are available in the GENESEQ™ database. The gene IDs and the corresponding patent accession numbers are listed in the first input file, as described in the 'methods' section.

The research area we chose is the development of antibodies for cancer treatment. In order to represent this area we have arbitrarily chosen the keywords: 'cancer', 'tumor', 'liver', 'over-expressed', 'antigen', 'vaccine', 'monoclonal', 'antibody', 'therapeutic', 'oncogene'. These keywords are listed in the second input file.

The idea here is to evaluate the IP position of these genes with respect to their application in the area of liver cancer therapeutic treatment with vaccine and/or monoclonal antibodies. In Fig. 1B, a subset of the output file describing part of the results obtained for gene IDs '41'and '109' is illustrated. A number of patent entries related to 'cancer' can be recognized and, among them, one entry containing the words 'therapeutic' and 'liver' can be identified. The user can then check the additional information displayed and, if necessary, can access the full patent entry by using the available hyperlink.

By using the 'PatentMatrixAggregate' script the 'standard' output can be then summarized in the 'aggregated' output file, as shown in Fig. 2. From this file it appears evident that the gene with ID 109 (ADCY3) is that which has the highest number of patent entries with cancer related keywords: 6 of them with the keyword 'vaccine', 2 with 'antibody' and 2 with 'therapeutic'. Obviously an accurate and in-depth study of the single patents related to each gene is needed for a sound assessment of the IP status of the genes. However, this preliminary overview already indicates that the gene ADCY3, in comparison to the others, is that with the greater likelihood of being protected from the IP standpoint in the areas of application explored.

## Conclusion

We have shown here that PatentMatrix efficiently explores the potential links between genes/proteins and patents, in a research area of interest defined by keywords. Being applicable to large lists, the tool is well suited to being adopted as part of a more extensive workflow of analysis (e.g. in a target identification/validation process). Although this semi-automatic approach does not and is not intended to substitute thorough patent evaluation by experts, it provides the user with a first-line straightforward and integrated overview of the often vast amount of existing context-specific patent literature. Finally, the systematic and structured mining of patents describing genes and proteins can also be exploited as a complementary resource to explore additional scientific information often not published in the literature.

## Authors' contributions

Armin Lahm has made substantial contributions to the conceptualization of the method and to data interpretation and drafting the manuscript. Emanuele de Rinaldis has conceptualized the method, implemented and tested the automated workflow and written the final version of the paper. Both authors read and approved the final manuscript.
